# The Hospital Officer's Bookshelf

**Published:** 1912-07-06

**Authors:** 


### THE HOSPITAL OFFICER'S BOOKSHELF.

The Doctors and the Insurance Act. By J. Dyer Bray. (Manchester: Thomas Griffiths and Co. Price 3d.)

In this little book a very clear and convincing statement is made of the medical man's case against the Act. The author is not himself a medical man, and claims on this ground that his conclusions are unbiassed. The "Six Cardinal Principles" embodied in the ultimatum of the British Medical Association has become a stock phrase in the reports of medical conferences which appear in the daily newspapers and elsewhere. To many who did not have the advantage of reading the original ultimatum the phrase is meaningless, and the reports, in consequence, fail to arouse the interest and sympathy they deserve. A very useful purpose is therefore served by the author, at the present juncture, in his explanation and justification of the "Six Cardinal Principles." It is manifestly of the greatest importance that a satisfactory agreement should be reached with the medical profession as to the administration of medical benefits under the Act. The insured are as much concerned as the doctors in this matter. It is to be hoped, therefore, that the book under review will obtain a wide circulation, and by enlightening public opinion will conduce to the end in view. The author also deals with the position of the voluntary hospitals under the Act, and clearly shows the necessity for modifying many of the provisions relating thereto.

Post-Mortems and Morbid Anatomy. By Theodore Shennan, M.D., F.R.C.S.E. Illustrated with photographs and coloured plates. (London: Constable and Co. 1912. Pp. 496. Price 18s. net.)

The average medical practitioner, it must be admitted, is not seen at his best when conducting a post-mortem examination, and his interpretation of pathological findings is sometimes characterised by a vagueness worthy of an ambassador. This is not to be ascribed to his own shortcomings, but simply to lack of opportunity for practice, and hence, when the occasion arises, he is glad to turn to some pathological expert or to consult some authoritative book on the subject. Dr. Theodore Shennan has added a most worthy manual to the literature on post-mortem work. Its text is clear and practical; abstruse theories and excessive details are omitted, or the reader is referred to an excellent bibliography. The general practitioner should find this book one eminently suitable for his needs. No doubt the influence of the Edinburgh School makes itself felt in the text, but the author's own large experience both at home and abroad insures a well-considered opinion on debateable points. On the actual technique of the operations involved Dr. Shennan is full and thoroughly practical; to follow his directions means the winning of half the battle. Many an examination has been rendered well-nigh fruitless by the havoc made by unskilful or faulty manipulations. We are glad to note the author lays stress on the importance of considering the size and weights of organs in relation to the height and weight of the individual; there is room for a considerable amount of research on this point. The order of the chapters follows more or less the usual order in which the post-mortem would be conducted, and the text is well supplied with very excellent illustrations, including a series of coloured plates. These are, without exception, all original and represent naked-eye appearances. A useful chapter is the one on the post-mortem changes produced by poisons; this should especially appeal to general practitioners. The author utters a warning, under the heading of "Chloroform," against regarding deaths really due to septic poisoning as being caused by delayed chloroform poisoning. In an appendix will be found fully described the most practically successful methods of preserving organs in their natural colours and the preparation of sections, etc. Dr. Shennan is to be congratulated on having compiled a text-book of great usefulness.

Technique of the Teat and Capillary Glass Tube and its Applications in Medicine and Bacteriology. By Sir A. E. Wright, M.D., F.R.S. (London: Constable and Co. 1912. Pp. 208. Price 10s. 6d. net.)

From small beginnings there has been evolved a technique which is almost overpowering in its ingenuity. As long ago as 1897 Sir Almroth Wright pointed out the advantages of the simple narrow bore pipette for measuring or diluting minute quantities of serum or blood, when this apparatus was equipped with a teat and marked with a blue grease-pencil, a air-bubble being admitted as an index. From such a simple affair has been elaborated a technique which is of wide and ever widening application, and which is fascinating even to those who are not laboratory experts. The need of a permanent record of this work as well as a manual of instruction for those who could not obtain access to competent teaching has long been felt, and in supplying this need Sir A. E. Wright has given full and overflowing measure to those who ask for his secrets. The book now so splendidly produced is one which will redound to the credit of both author and publisher. In a work of this description the illustrations must be of the greatest importance, and here we find them in most helpful profusion. Some of the coloured plates, too, are beautiful examples of the possibilities of artistic reproduction. As to the text itself, we can only express admiration at the way in which the worker is taken step by step through the various stages of pipette making, and the manipulation of glass tubing to the intricacies of serological experiments. Full and very clear expositions are given of the methods of examining the blood for various purposes, such as its calcium content, coagulation time, antitryptic power, and its bacteriotropic reactions. The book has been compiled with obvious care, but yet the figures relating to certain quantitative solutions lack that accuracy which would seem to become second nature to a follower of Sir A. E. Wright. Naturally the subjects of opsonic measurements and the application of the technique to the Bordet-Gengou reactions, as well as the preparation of vaccines, receive full attention. The book is one of first-class importance, and one which will, as the author says, appeal to "two different classes of laboratory workers: to the man who wants a ready-made technique for measuring this or that function of the blood, and wants nothing more; and to the research worker who looks not only to applications of the technique already made, but to the further applications which will follow, now that science is flooding everywhere into medicine." That this technique will in the near future find further applications and undergo considerable development, is an opinion which no scientific medical worker will be disposed to disagree with.

Sprue: Its Diagnosis and Treatment. By Charles Begg, M.B. (Bristol: J. Wright and Sons. 1912. Pp. 124. Price 6s.)

This monograph by an author who has spent many years in practice in China is an *ex parte* statement of the case for a particular method of treating sprue. The method was hit upon empirically, but the statistics which Dr. Begg quotes do seem to show that it is effective, and lend some considerable colour to his hypothesis as to the pathology of the disease. More than that, it is not possible to say as yet, though it may be expected that the Commission now investigating sprue will test this and any other plausible line of treatment and causation. Briefly, Dr. Begg regards santonin as a specific remedy for sprue; with this qualification, that only yellow santonin is efficacious. Pure new santonin is white, but this variety is useless for sprue; on six months' exposure to tropical sunlight it goes yellow, and certain of its polarimetric qualities are changed. The older and yellower the drug, the better it acts; and this holds good, according to Dr. Begg, both for its vermifuge and for its sprue-curing properties. The dose advocated is five grains, night and morning, well rubbed up in a teaspoonful of olive or almond oil. This quantity can be given for months, if necessary, without danger. Stress is also laid upon the treatment of secondary symptoms by stomach lavage and drugs.

